# Estimating the economic impact of a possible equine and human epidemic of West Nile virus infection in Belgium 

**DOI:** 10.2807/1560-7917.ES.2016.21.31.30309

**Published:** 2016-08-04

**Authors:** Marie-France Humblet, Sébastien Vandeputte, Fabienne Fecher-Bourgeois, Philippe Léonard, Christiane Gosset, Thomas Balenghien, Benoît Durand, Claude Saegerman

**Affiliations:** 1 University of Liege, Liege, Belgium; 2 University Hospital, Liege, Belgium; 3 French Agricultural Research Centre for International Development (CIRAD), Montpellier, France; 4 French National Institute for Agricultural Research (INRA), Montpellier, France; 5 University Paris Est, French Agency for Food, Environmental and Occupational Health & Safety (ANSES), Maisons-Alfort, France

**Keywords:** West Nile virus, cost analysis, scenario analysis, strategic vaccination, hospital costs, productivity lost

## Abstract

This study aimed at estimating, in a prospective scenario, the potential economic impact of a possible epidemic of WNV infection in Belgium, based on 2012 values for the equine and human health sectors, in order to increase preparedness and help decision-makers. Modelling of risk areas, based on the habitat suitable for *Culex pipiens*, the main vector of the virus, allowed us to determine equine and human populations at risk. Characteristics of the different clinical forms of the disease based on past epidemics in Europe allowed morbidity among horses and humans to be estimated. The main costs for the equine sector were vaccination and replacement value of dead or euthanised horses. The choice of the vaccination strategy would have important consequences in terms of cost. Vaccination of the country’s whole population of horses, based on a worst-case scenario, would cost more than EUR 30 million; for areas at risk, the cost would be around EUR 16–17 million. Regarding the impact on human health, short-term costs and socio-economic losses were estimated for patients who developed the neuroinvasive form of the disease, as no vaccine is available yet for humans. Hospital charges of around EUR 3,600 for a case of West Nile neuroinvasive disease and EUR 4,500 for a case of acute flaccid paralysis would be the major financial consequence of an epidemic of West Nile virus infection in humans in Belgium.

## Introduction

West Nile virus (WNV) is a vector-borne pathogen, member of the genus *Flavivirus* (family *Flaviviridae*); its main vectors are mosquitoes belonging to the *Culicidae* family, genus *Culex* [1,2]. The infection is maintained in a bird–mosquito enzootic cycle, and birds, especially passerines, are the primary reservoir hosts. Horses and humans are considered as accidental dead-end hosts, and are thought not to transmit the virus to other mosquitoes [3]. The disease generates clinical signs mainly in horses and humans, while most infected birds in Europe are not clinically affected [4]. The majority of horses remain asymptomatic, and about 10% of clinical cases develop neurological signs [5]. In humans, after an incubation period of 2 to 14 days, two main clinical pictures can be observed: an influenza-like syndrome (West Nile fever, WNF) and a neuroinvasive form (West Nile neuroinvasive disease, WNND) [4].

The virus is considered as an emerging pathogen in numerous parts of the world. In the United States (US), it has been responsible for substantial socio-economic losses, both in the equine industry and in the human health sector, since its emergence in 1999 [6]. In Europe, the virus is constantly expanding its geographical distribution [7] and even re-emerging in some areas: indeed, several equine cases have been reported since September 2015 in France, which also registered its first autochthonous human case since 2003 [8]. To date, no autochthonous human case has been reported in Belgium, but the first imported case was described in 2013, an elderly woman who had travelled to Greece [9]. As *Cx. pipiens*, a common mosquito vector of WNV in Europe [10,11], is endemic in Belgium, the risk of emergence in the near future should be seriously considered. There is thus a need to get prepared in advance of such an emergence, in terms of management strategies and their respective socio-economic impact. Indeed, western Europe has a recent history of severe economic losses associated with animal diseases, e.g. with bluetongue disease epidemics in 2006 [12] and Schmallenberg virus disease in 2011–12 [13]. 

The objective of the study presented here was to estimate, in predictive scenarios, the economic impact on both the equine and human health sectors of the spread of WNV in Belgium during an epidemic. When dealing with an unpredictable occurrence of a disease (limited knowledge about likelihoods) but with good knowledge of outcomes, a scenario analysis is recommended [14,15]. Estimating the cost of an illness is a useful aid to policy decision-making. It identifies the different components of cost and the size of the contribution of each sector, which can help determining mitigation measures, research and funding priorities by highlighting areas where inefficiencies may exist and savings could be made [16]. 

## Methods

### Determination of risk areas and populations at risk

In order to determine the proportion of Belgian territory representing a habitat suitable for *Cx. pipiens* (the main potential vector for WNV in Belgium), land cover data were extracted from the CORINE (Coordination de l’information sur l’environnement) land cover (CLC) database [17]; suitability of different land covers was further determined for *Cx. pipiens* and the proportion of these suitable land covers was estimated at the district level [18]. This first step allowed us to determine the equine (using information from the Belgian Horse Confederacy) and human [19] populations at risk, at the district level. It was assumed that the density of WNV-competent birds was high and homogeneously distributed across the whole territory. The distribution of equestrian centres at district level was used to estimate their potential loss of earnings as a result of an epidemic; data were provided by regional and provincial equestrian leagues.

### Equine industry

In Belgium, contrary to what is observed in many parts of the US, such as Texas, Colorado or Nebraska [20], horses are mostly used for recreational purposes, and their agricultural importance is very limited. A 2010 study assessed the economic weight of equine industry in southern Belgium and distinguished three categories of horses [21]: (i) high-value horses (20%), mostly show horses that are cared for intensively; (ii) leisure horses (40%), which usually spend the winter indoors and the summer in pastures (horses of equestrian centres also fall into this category); and (iii) semi-feral horses (40%), which spend most of their time in pastures, and are occasionally used for leisure (they are owned by individuals but are often untrained).

Economic impacts of WNV on the equine sector were estimated on the basis of the disease characteristics observed in previous European epidemics of WNV infection. Two scenarios based on infection rates previously estimated during outbreaks in France were included in the model: 8.5% infection rate [22] vs 34% infection rate [23]. A 10% morbidity rate was assumed, according to French data [5], and the number of equine cases was determined as follows:

N_horse cases_ = [a × 0.1 × b × c]

a = infection rate = 8.5% vs 34% (proportion of horses infected by the virus, but not necessarily showing clinical signs) [22,23];

0.1 = morbidity rate (10% of infected horses will develop symptoms of disease) [5];

b = proportion of the district (in terms of land cover) suitable for *Cx. pipiens* (used to determine the whole population of horses living in risk areas);

c = district total horse population.

The hospitalisation rate of neurological equine cases was fixed at 35% of clinically affected horses [24], and a 28% case fatality rate, as observed in France, was applied [25]. The mean length of the clinical disease was considered to be seven days (as was duration of hospital stay) [26]. The duration of an epidemic was estimated to be 2.5 months, with the first case reported on 1 August and the last case resolved on 21 October. This is in agreement with findings of most European equine cases, which are reported between August and October [27]. 

In terms of Belgian legislation on animal movement in case of epidemics of WNV infection, restrictions only apply for suspected and confirmed cases of WNF, which cannot be moved, except to be transported to a veterinary healthcare facility [28].

A distinction was made between non-hospitalised and hospitalised horses to estimate economic costs. The following costs were included for non-hospitalised horses: visits of a veterinary practitioner, serological diagnosis (ELISA) and reverse transcription (RT)-PCR, as well as treatment (non-steroidal anti-inflammatory medicine). Hospitalised horses were considered to be seen first by a veterinary practitioner before being referred to a veterinary hospital. The estimation of costs included also: costs of a seven-day stay, complementary examinations (blood analysis, radiography, puncture of cerebrospinal fluid, diagnosis and neurology) and seven-day treatment (steroidal and non-steroidal anti-inflammatory medicine, intravenous fluids) [24]. For horses that died or had to be euthanised, transport and destruction of cadavers, as well as replacement value, were considered for the three categories of horses (i.e. high-value, leisure and semi-feral).

The cost of vaccination was also included in the estimations. According to current Belgian legislation, the vaccination of equids against WNV is not mandatory; nevertheless, the Minister of Agriculture could modify that decision in case of epidemics [29]. Two vaccination scenarios were thus applied in our model in order to investigate the potential impact of such a preventive measure on the estimation of costs: the first scenario relies on the vaccination of the entire equine population (except sick horses), while the second scenario considered the vaccination of horses in risk areas only. Primary vaccination consists of two doses, the second dose being administered 3–5 or 4–6 weeks later, depending on the vaccine used; indeed, in Belgium, two vaccines are registered for horses older than 5–6 months [30].

According to the Belgian Federal Authorities, an outbreak is confirmed when there is proof that the WNV is effectively transmitted by a local infected vector population, competent for transmitting the virus [28,29]. Consecutively, passive clinical surveillance is enhanced and active serological surveillance is implemented for horses in the area where the epidemiological investigation is carried out (within a 50 km radius) [28,29]. Active surveillance in horses consists of an ELISA (detection of IgG and IgM) performed on blood samples, by the National Reference Laboratory in Brussels. In order to determine the number of blood samples that would need to be taken, estimation was made through Win Episcope 2.0 software, considering a sample size needed to detect the disease, with a 5% expected prevalence and 95% confidence interval. For equestrian centres, serology was also performed on asymptomatic horses located in the same centre, as suggested in the WNV scenario elaborated by the Belgian Federal Agency for the Safety of the Food Chain [28]. Active surveillance was considered to be implemented throughout the epidemic (13 weeks), with a 15-day frequency [31], which means six sampling periods.

In Belgium, any movement of a live animal suspected to have viral encephalitis is prohibited [29]. As it is also mandatory to isolate horses that are suspected or confirmed to have WNV infection [29], associated additional costs of feed (cereals, hay and water) and litter (straw) were estimated for the duration of the epidemic for all equine cases (hospitalised and non-hospitalised horses), because suspected cases are assumed not to be left out on pastures.

The loss of income for affected equestrian centres (mean of 20 horses for public use per centre [21]), of the 830 registered in the country, was also estimated, considering the mean number of days of lost-use for equids clinically sick and recovering [32]. All assumptions made in our study are compiled in Table 1.

**Table 1:** Model parameters for estimating economic impact of an epidemic of West Nile virus infection in Belgium, based on 2012 values

Parameter	Value	Unit	Source
**Vectors**			
Proportion of territory (land cover) representing a habitat suitable for *Culex pipiens*	Variable	%	[18]
Duration of the epidemic	2.5	Months	[7]
**Horses**			
District horse population	Variable	Number	Belgian Horse Confederacy, Jean-Pierre Devos, personal communication, July 2013
Equestrian centres (per district)	Variable	Number	Belgian Regional and Provincial Equestrian leagues, Jan Deboitselier, personal communication, July 2013
Horse infection rate (proportion of the horse population living in the risk areas infected by the virus)	8.5 vs 34	%	[22,23]
Horse morbidity rate (will develop clinical signs of disease)	10	%	[5]
Hospitalisation rate for neurological cases	35	%	[24]
Horse case fatality rate (mortality among neurological cases; the most severe cases being hospitalised)	28	%	[25]
Mean length of the clinical disease (duration of hospital stay for hospitalised horses)	7	Days	[26]
Active surveillance (screening)	ELISA results	NA	[28,29]
Duration of active surveillance (whole epidemics)	13	Weeks	[28]
Frequency of sampling – active surveillance	15	Days	[31]
Detection of the disease – 5% expected prevalence (95% confidence interval)^a^	Variable	Number	Win Episcope 2.0; [24]
Mean number of horses per equestrian centre (for public use; not privately owned)	20	Number	[21]
**Public health**			
District human population	Variable	Number	[19]
Human infection rate (proportion of the population living in the risk areas infected by the virus)	2 vs 15	%	[36]
Human morbidity rate for WNND (all patients assumed to be hospitalised)	0.7	%	[37]
Proportion of AFP among WNND cases (all assumed to be > 65 years-old)	3	%	[39]
Human case fatality rate (mortality among patients with WNND)	11	%	[7]
Mean age of deceased patients	78	Years	[58]
Mean hospitalisation length of stay	9	Days	Philippe Leonard, personal communication, July 2011
Home recovery			
Duration (working days)	20	Days	Philippe Leonard, personal communication, July 2011
Daily cost for a home nurse (two visits a day – one hour a day)	16	Euros	[59]
Daily cost for a caregiver (eight hours a day)	62	Euros	[60]
Productivity lost			
Percentage of men in the population	49.06	%	[19]
Percentage of women in the population	50.94	%	[19]
Activity rate^b^ in people aged 15–64 years – men	72.5	%	[19]
Activity rate^b^ in people aged 15–64 years – women	61.3	%	[19]
Employment rate^c^ – men	66.9	%	[19]
Employment rate^c^ – women	56.8	%	[19]
Proportion of employees^d^	62.0	%	[19]
Proportion of workers^d^	38.0	%	[19]
Mean annual growth (to adapt 2004 healthcare prices^e^ to 2012 values)	1.7	%	[42]
Costs associated with the death of a patient (insurance claims paid to beneficiaries)	9,800	Euros	Belgian insurance company, personal communication, November 2014
Mean occupational interruption to estimate productivity lost (working days)	30	Days	[40]
Mean gross monthly salary – Men (2012) – Employee	3,668	Euros	[19]
Mean gross monthly salary – Men (2012) – Worker	2,749	Euros	[19]
Mean gross monthly income – Men (2012) – Self-employed	3,700	Euros	[19]
Mean gross monthly salary – Women (2012) – Employee	3,372	Euros	[19]
Mean gross monthly salary – Women (2012) – Worker	2,527	Euros	[19]
Mean gross monthly income – Women (2012) – Self-employed	3,413	Euros	[19]

AFP: acute flaccid paralysis; NA: not applicable; WNND: West Nile neuroinvasive disease.

^a^ The decision to select 5% expected prevalence arose from the results of Durand et al. in France [22] who estimated an 8.5% prevalence in a West Nile virus outbreak in horses in 2000; thus, a 5% threshold seemed realistic.

^b^ Active individuals include all persons of working age, who carry out a paid activity (population active occupied) and job seekers (population active unoccupied/unemployed) [61].

^c^ We considered unemployed people not only to be those who did not work (and were entitled to unemployment benefit), but also people not on the labour market ,such as housewives/house husbands.

^d^ We made a distinction between employees (who generally carry out intellectual work) and workers (who mainly carry out manual tasks).

^e^ All information regarding costs for a hospitalised case was obtained from hospital records regarding a patient hospitalised in Belgium for WNND in 2004: we therefore adapted the costs to 2012 values, taking into account the evolution of the consumer price index.

### Public health

In humans, two main clinical disease forms are described: WNF and WNND [33]. Our study only included patients affected by WNND for the estimation of costs. 

The potential consequence of WNF would logically be for the affected person to visit to a general practitioner (GP) and miss five days at work [34]. It seems unlikely, however, that all affected people would consult their GP. The impact on productivity should be limited, as these people would not be replaced for such a short length of absence, and they would have to deal with the backlog on returning to work. Alternatively, many people with WNF could miss work for less than five days (e.g. self-employed people who lose a net salary when missing a working day). 

Three main syndromes may be observed in case of neuroinvasive disease: meningitis, encephalitis and acute flaccid paralysis (AFP) [4]. As a previous study estimated the costs for meningitis and encephalitis to be quite similar [35], our work distinguished between two main syndromes: meningoencephalitis and AFP.

The human population at risk was determined per district according to the same procedure as for equids. Two scenarios were tested, based on infection rates estimated during an equine WNV outbreak in southern France in 2000 [36].

The number of WNND cases was calculated as follows:

N_WNND cases_ = [a × b × c × 0.007]

a = district total population;

b = proportion of the district (in terms of land cover) suitable for *Cx. pipiens*;

c = infection rate (2%, as estimated among 1,104 blood donors living outside the region of the 2000 equine epidemic of WNV infection in southern France, or 15%, determined among 1,053 blood donors living in the epidemic zone [36]);

0.007 = percentage of patients develop WNND [37].

A 0.7% morbidity rate for WNND was considered, which means that among infected patients, 0.7% will develop the neuroinvasive form requiring hospitalisation [37]. Infection rates were assumed to be uniformly distributed in the population, without considering criteria such as age and sex, as they have not been identified as risk factors in an important case–control study performed in Greece in 2010 [38]. Of 197 WNND cases reported in Greece in 2010, 3% were classified as AFP cases [39]. An 11% case fatality rate was applied, as reported for the whole of the European Union (EU) in 2012 [7]. All WNND cases were assumed to be hospitalised, as considered in a previous work [40]. The mean hospitalisation length of stay was nine days (Philippe Leonard, personal communication, September 2011). No vaccine is currently available for humans and the only treatment is supportive care [4]. All assumptions made in our study are shown in Table 1.

As for the equine sector, economic impacts were estimated taking into account several aspects, such as medical and hospital costs, costs for home care, compensation paid for the death of a patient and costs associated with work absenteeism. Short-term initial costs included a visit to a GP. In Belgium, the healthcare system is divided into state and private sectors. It is funded by a combination of social security contributions and health insurance funds. Healthcare insurance is mandatory: patients generally pay the costs and are then reimbursed for part of the charges. Individuals can also improve their cover by taking out private insurance, which allows them to be fully refunded for all medical costs [41]. Inpatient costs for acute care and rehabilitation were also considered: hospital stay (room and board charges), complementary tests and examinations (e.g. electroencephalogram, cerebrospinal fluid analysis, visit of a neurologist, heart monitoring, imaging, laboratory investigation and serology) and pharmacy/medical supplies (treatment charges: pharmacy, drugs such as antibiotics and antiviral medicine, and anti-epileptics, injections, medical supplies, intravenous fluids and intravenous therapy). 

Costs related to hospital charges were indexed, as information was collected from hospital records concerning a patient hospitalised in Belgium for WNND in 2004 (encephalitis with epilepsy) (Philippe Leonard, personal communication, September 2011). We estimated the costs in 2012: thus a mean annual growth rate of 1.7% was applied to adapt healthcare prices to the evolution of the consumer price index for the eight-year period and convert them into 2012 values [42]. Hospitalisation costs were estimated to be 1.25 times higher for AFP cases than the costs for patients affected by WNND, based on estimates used in a study in the US [35].

Costs associated with the death of patients were estimated through an ex post approach, as part of human capital theory [43], and consisted of insurance claims paid to beneficiaries. In order to obtain representative data, a directory of accidents from a car insurance branch of a Belgian insurance company was analysed over a five-year period (2009–13). The insurance claim considered only covered the economic loss, and not the suffering of close relatives (i.e. pretium doloris). Considering a 9.78% annuity conversion rate, estimated according to the life expectancy of a 78 year-old person, the insurance claim reached EUR 9,800, on the basis of a 1.5% annual discount rate.

Additionally, a mean occupational interruption of 30 working days (for symptomatic disease and recovery) was taken into account to estimate productivity lost [40]. To establish the distribution of patients in different employment categories, statistics relative to the labour market – such as employment rate according to age and sex, status of working patients (self-employed vs employed) and mean gross monthly wages – were provided by the Belgian Federal Public Service of Economy, Small and Medium Enterprises, Self-employed and Energy, for 2012. We made a distinction between employees (who generally carry out intellectual work) and workers (who mainly carry out manual tasks). 

## Results

**Figure 1:**
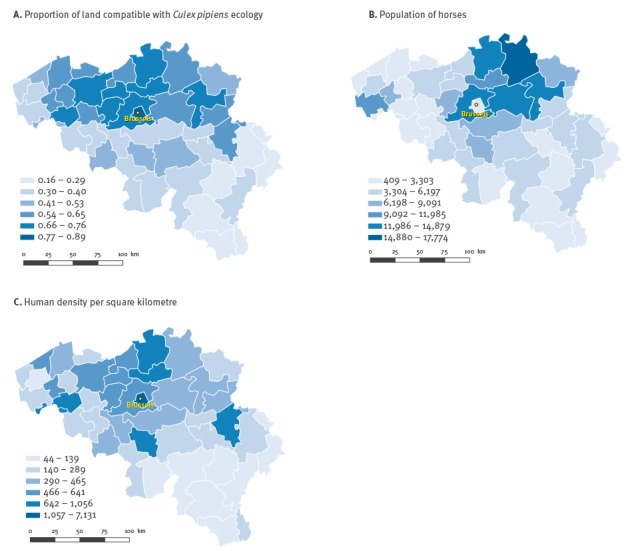
Proportion of land compatible with *Culex pipiens* ecology (A), horse population (B) and human density per km^2^ (C), per district in Belgium, 2012

Habitat suitability in Belgium for *Cx. pipiens* is illustrated at the district level in Figure 1 (panel A). The proportion of suitable habitat was the basis for estimating the number of individuals at risk (in terms of proportions of the whole population of the district). Horse and human populations are shown in panels B and C, respectively.

### Equine industry

Estimates of economic costs associated with WNF per horse are summarised in Table 2.

**Table 2:** Economic losses and costs associated with West Nile virus fever in the equine industry following an epidemic of West Nile virus infection in Belgium, estimated per horse, 2012 values

Economic impact	**Value in euros**
Disease in horse	
Visit of veterinary practitioner	42
Hospitalisation (7 days’ duration)^a^	
Stay of the horse	69
Veterinarian specialists (internal medicine, neurology)	85
Complementary examinations (blood sampling and analysis, X-rays, CSF puncture and analysis)	191
Medical treatment (NSAID, SAID, supportive treatment)	396
No hospitalisation	
Medical treatment (NSAID)	27
Diagnosis (serology, RT-PCR)	76
Indirect costs – containment of cases in stables	
Extra feed	
Hospitalised surviving horse^b^	
High-value horse	33
Leisure horse	19
Semi-feral horse	12
Non hospitalised horse^c^	
High-value horse	39
Leisure horse	22
Semi-feral horse	14
Extra bedding	
Hospitalised surviving horse^b^	
High-value horse	44
Leisure and semi-feral horse	22
Non-hospitalised horse^c^	
High-value horse	53
Leisure and semi-feral horse	26
Management of horse mortality	
Transport, destruction of cadaver	70
Replacement value for dead/euthanised horse	
High-value horse	10,000
Leisure horse	4,000
Semi-feral horse	2,000
Loss of earnings	
Per affected horse for rent^d^	1,638
Vaccination	
Two doses of vaccine, veterinary costs	144

CSF: cerebrospinal fluid; NSAID: non-steroidal anti-inflammatory drug; RT: reverse transcription; SAID: steroidal anti-inflammatory drug.

^a^ Hospitalised horses were assumed to be the most severely affected, when considering nervous clinical signs.

^b^ Mean income per horse (39 workdays during clinical disease and recovery; three hours of work a day and one day off per week).

^c^ A 42-day recovery period was considered for a horse that was not hospitalised.

^d^ Regarding indirect costs and containment of cases indoors, a duration of 35 days was considered for recovery of a horse after its discharge from a veterinary hospital.

When considering national estimations (Table 3), the main costs would be related to the replacement of dead or euthanised horses, followed by hospitalisation charges. If vaccination was implemented (whole territory or areas at risk), associated costs would then represent the major expense.

**Table 3:** Economic losses and costs associated with West Nile virus fever in the equine industry following an epidemic of West Nile virus infection in Belgium, by infection rate scenario, estimated at national level, 2012 values

Economic impact	8.5% infection rate scenario		34.0% infection rate scenario	
	Cost in euros	Number	Cost in euros	Number
General				
Hospitalisation (7 days)	278,748	356	1,116,558	1,426
No hospitalisation	145,640	662	582,560	2,648
Diagnosis (serology, RT-PCR)	77,368	1,018	309,624	4,074
Management of cadavers	7,000	100	27,930	399
Active surveillance^a^ (serology)	147,888	24,648^b^	147,888	24,648^b^
Containment of cases indoors – maintenance costs	47,118	918	188,564	3,674
Replacement value				
Total	440,000	100	1,750,000	399
High-value horses	200,000	40	790,000	79
Leisure horses	160,000	40	640,000	160
Semi-feral horses	80,000	20	320,000	160
Total (general and replacement value)	1,143,762	NA	4,123,124	NA
Loss of earnings for equestrian centres				
Amount	113,022	69	450,450	275
Vaccination costs (2 doses of vaccine, veterinary costs)				
100% coverage	33,091,632	229,803	32,651,712	226,748
Areas at risk	17,105,040	118,785	16,665,264	115,731

NA: not applicable; RT: reverse transcription.

The population of horses was subdivided into three categories: 20% high-value horses, 40% leisure horses and 40% semi-feral horses [21]. 

^a^ Expected 5% prevalence (set at this level to be realistic compared with that in [22]) determined according to the equine population per district (risk areas)). Six sampling periods would be implemented, with sampling planned every two weeks [31]. If a horse is affected in a rental centre, all other animals should be regularly tested as well (mean of 20 horses per rental centre) [21].

^b^ Number of tests performed. 

At national level, the lost revenue for equestrian centres following an outbreak would reach between EUR 113,022 and EUR 450,450 for the 8.5% and 34% infection rate scenarios, respectively, assuming that two clinically sick horses per equestrian centre were not used during the time of clinical disease/treatment and recovery. The occurrence of WNV infection in these equestrian centres also implied the sampling of all horses located in the same centre (mean of 20 horses per centre). Detailed estimations can be consulted in supplementary material [44-47].

### Public health aspects

**Figure 2:**
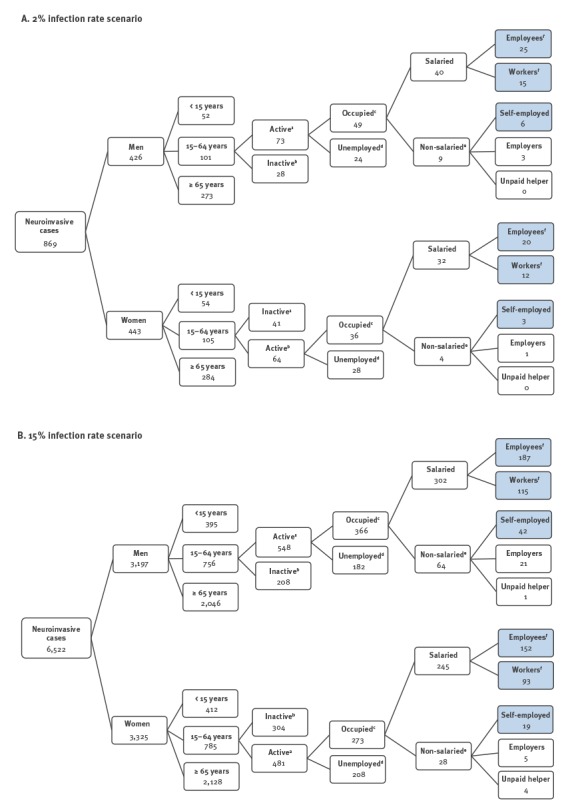
Estimated number of patients with West Nile neuroinvasive disease, by employment category and infection rate scenario, following an epidemic of West Nile virus infection in Belgium, 2012

The estimated distribution of WNND cases, for both infection rate scenarios, according to the criteria used is shown in Figure 2. No productivity loss was estimated for people aged over 65 years and for patients with AFP, as they are all assumed to be retired. Nor did we estimate productivity lost for caregivers (often another family member), who might miss work to care for the recovering patient after hospital discharge. Home care costs were nevertheless estimated for surviving hospitalised patients during their recovery (20-day period).

These estimates were based on statistics related to the labour market, e.g. employment rate (according to sex or status) of working patients. Costs and losses were not estimated for children under 15 years of age and students.

Shaded boxes show the number of patients estimated for each professional category considered in our estimations of costs (related to productivity losses).

^a^ Active individuals include all persons of working age, who carry out a paid activity (population active occupied) and job seekers (population active unoccupied/unemployed) [61].

^b^ Inactive individuals include all persons, even those under 15 years of age, who are not economically active [61].

^c^ Occupied individuals include all persons, aged 15 years and above, who have a paid job (self-employed, employees and workers).

^d^ We considered unemployed people not only to be those who did not work (and were entitled to unemployment benefit), but also people not on the labour market, such as housewives/house husbands.

^e^ In the ‘non-salaried’ category, only self-employed people were considered for the estimate of losses. Indeed, no losses can be estimated for unpaid helpers; losses for employers were those estimated for occupied people in our study.

^f^ We made a distinction between employees (who generally carry out intellectual work) and workers (who mainly carry out manual tasks).

The costs incurred per patient with WNND, as well as the associated productivity losses, are summarised in Table 4.

**Table 4:** Economic losses and costs of West Nile neuroinvasive disease in humans following an epidemic of West Nile virus infection in Belgium, estimated per human case, 2012 values

Economic impact	Cost in euros
Initial visit, to a general practitioner	23
Diagnostic tests (serology)	21
Hospitalisation: WNND case	
Total hospitalisation cost	3,553
Stay^a^ (9 days hospitalisation)	739
Pharmaceutical expenses	926
Provision of services^b^	1,888
AFP case^c^	
Total hospitalisation cost	4,441
Home care^d^ (during a 30-day home recovery period)	
Costs	2,340
Productivity lost due to work absenteeism	
Men	
Employee^e^	5,502
Worker^e^	4,124
Self-employed	5,550
Women	
Employee^e^	5,058
Worker^e^	3,791
Self-employed	5,120
Compensation	
Paid to beneficiaries after the death of a patient^f^	9,800

AFP: acute flaccid paralysis; WNND: West Nile neuroinvasive disease.

^a^ Includes costs of hospital stay, medical services, patient’s share of the costs, initial clinical tests and daily pharmacy costs.

^b^ Includes monitoring of vital functions, medical imaging, clinical biology, neurology, etc.

^c^ AFP occurs in 3% of all WNND cases [39]. Hospitalisation costs are 1.25 higher for AFP cases compared with those for WNND cases [35].

^d^ Including costs for a nurse and caregiver. Home care costs are underestimated for AFP cases, as recovery can take several months [62].

^e^ We made a distinction between employees (who generally carry out intellectual work) and workers (who mainly carry out manual tasks).

^f^ Estimated from compensation paid to beneficiaries by a Belgian insurance company following the death of a 78 year-old person in a car accident (median age of patients who died from WNND being 78 years).

National estimates, as shown in Table 5, highlight the importance of hospital costs (around 50% of total costs), compared with those for home care during recovery (about 30% of total costs). Detailed estimations can be consulted in supplementary material [44-47].

**Table 5:** Economic costs and productivity losses of West Nile neuroinvasive disease in humans following an epidemic of West Nile virus infection in Belgium, by infection rate scenario among people at risk, estimated at national level, 2012 values

Economic impact	2% infection rate scenario		15% infection rate scenario	
	Cost in euros	Number of patients	Cost in euros	Number of patients
Visits to a general practitioner	19,987	869^a^	150,006	6,522^a^
Hospital charges				
Total	3,110,645	869^a^	23,346,714.00	6,522^a^
WNND	2,995,179	843^b^	22,476,278	6,326^b^
AFP	115,466	26^c^	870,436	196^c^
Other				
Diagnosis (serology)	18,249	869^a^	136,962	6,522^a^
Home care (30-day recovery period after hospital discharge)	1,808,820	773^d^	13,583,700	5,805^d^
Work absenteeism (productivity losses)	495,924	120^e^	2,857,613	587^e^
Compensation paid to beneficiaries (after the death of a patient)	940,800	96^f^	7,026,600	717^f^
**Total **	**6,394,425**	869^a^	**47,101,595**	6,522^a^

AFP; acute flaccid paralysis; NA: not applicable; WNND; West Nile neuroinvasive disease.

AFP occurs in 3% of all WNND cases [39]. Hospitalisation costs are 1.25 higher for AFP cases compared with those for WNND cases [35].

^a^ Number of patients consulting a general practitioner (WNND and AFP cases).

^b^ Number of patients hospitalised for WNND.

^c^ Number of patients hospitalised for AFP.

^d^ Number of hospitalised patients who survived and needed homecare after discharge from hospital.

^e^ Number of hospitalised patients, who are active on the labour market (and have a job) and for whom losses due to work absenteeism were estimated.

^f^ Number of deceased patients.

## Discussion

We estimated, in a prospective scenario, the economic impact of a possible epidemic of WNV infection in Belgium, according to different infection rate scenarios in horses and humans. Considering the whole territory would be concerned might be an overestimation (worst-case scenario). In view of epidemics reported in the EU to date, spatial extension might be less pronounced. All conditions for epidemic spread are met in the northern part of the country, as *Cx. pipiens* habitat and areas of human density are closely related, as are areas with horse populations. Our selected infection rates might seem low compared with those in WNV-endemic areas of Africa, where seroprevalence of more than 90% was detected in horses in some places [48], but they reflect better the dynamics of the virus in Europe. Applying infection rates reported in epidemic situations allowed us to identify substantial costs and losses associated with the disease, both in horses and humans.

### Impact on the equine industry

The total estimated cost for treating a horse affected by neurological disease due to WNV in 2012 would be more than estimated in the US, in Colorado and Nebraska, in 2002 [32]. Intensive care for moderate cases (wobbly gait, difficulty eating, signs of colic, reluctance to move, hypersensitivity to noise and touch, and altered awareness) was estimated to cost USD 400 (EUR 381) (equivalent to USD 539 (EUR 408) in 2012). For hospitalised horses showing severe neurological disease, we estimated such costs to reach EUR 741 (USD 917). Our estimates are more in line with a later survey performed in Texas, US, for 2002, in which veterinarians estimated the cost of treatment of a severe WNND equine case to be over USD 701 (EUR 668) (when considering a mean annual growth rate of 1.7%, as mentioned above) [49].

Vaccination is the main cost associated with epidemic spread of WNV in horses: the strategy, if implemented, would have a major economic impact, as vaccinating the whole equine population (100% coverage scenario) would cost over EUR 30 million. The time needed to produce and commercialise enough vaccine doses would not be less than six to nine months. Thus we assume that complete vaccination of the whole population of horses could be achieved by the following vector season. The vaccine should be administered to horses aged five to six months and above [30]: unfortunately, the proportion of animals falling into this age category was not available, which would have allowed us estimate more precisely the costs. Thus the vaccination costs detailed here are probably overestimates (based on a worst-case scenario).

A recommendation to keep horses indoors would not be very effective against *Cx. pipiens* if additional measures such as mosquito nets or fans are not installed, as these mosquitoes are also active indoors. Nevertheless, from a practical point of view, it is probable all horse owners would not have the possibility or willingness to follow such a recommendation and to invest in adequate protective equipment. Furthermore, once horses are completely vaccinated, such a recommendation would no longer be relevant. The lost revenue for equestrian centres, due to affected horses not being used during the time of clinical disease/treatment and recovery, is very similar to the losses Gavlan et al. estimated in Texas, US, for 2002 [49]. 

As a prospective study, our estimations have, of course, some limitations. Several aspects were not taken into account to estimate economic impact, such as: (i) the costs of surveillance in birds (wild avifauna and sentinel domestic poultry), as it has already been implemented since 2010, and would not generate additional costs [50]; (ii) entomological surveillance, as this has not been clearly defined in the Belgian context to date; (iii) the preventive action of applying repellents, as this is suspected to be difficult to implement in the field [51] as people’s perceptions and behaviour towards protecting themselves and their horses is unknown; and (iv) impact of epidemic spread of WNV on trade of horse semen, even though the World Organisation for Animal Health (OIE) recommends that its member states should not impose trade restrictions on dead-end hosts such as horses [52]. In addition, some horse owners might have their horses insured, particularly if they are high-value horses: that parameter was not considered in our study due to lack of data.

### Impact on public health

Regarding the economic consequences for the human health sector, our estimations of hospital costs (EUR 3,553 for a WNND case and EUR 4,441 for an AFP case) are less than USD 8,274 (EUR 7,890), which was the median cost of inpatient treatment calculated for a WNV disease epidemic in Louisiana, US, in 2002 [53], and far below the USD 33,143 (EUR 28,094) estimated by Barber et al. in California, US, in 2005 [40]. In our study, several criteria were not taken into account, such as residual neurological after-effects. 

Even if a diagnostic test would be performed systematically for WNND patients, results of analyses arrive late, sometimes after hospital discharge (Philippe Leonard, personal communication, July 2011). Furthermore, confirmation of diagnosis would not provide an alternative to effective palliative medical care.

It is not surprising that hospital costs were higher than productivity losses, as more elderly patients develop neuroinvasive disease [4]; no productivity loss was estimated for people aged 65 years and above, as they are not considered as part of the labour market any more. Home care of recovering patients was the second most expensive cost. It is important to note that, in Belgium, professional home care services are partly refundable through the healthcare system, so family members are not systematically obliged to miss work to provide care to the patient. They can turn to these professionals to take care of them. Nevertheless, it was decided to include home care costs in our estimations. 

Our WNND scenario was based on estimates related to the overall population of areas at risk, infection rate and morbidity rate for WNND. Some cases could pass unnoticed at the beginning of the epidemic. An mean nine-day hospitalisation stay was selected, which is slightly longer than the length reported in a five-year survey of initial and long-term medical and lost-productivity costs for patients hospitalised in Colorado, US, in 2003 [35]. Higher costs are expected for AFP cases, due to longer hospitalisation and a higher lost productivity [35].

As for the equine part of the study, our estimates for the public health section have several limitations, as the study was performed prospectively. The control of vectors should be recommended to individuals and to public health authorities in case of a severe epidemic, but associated costs were not included in the estimations, in contrast to a previous study [35]. We did not consider impact of preventive use of chemical repellents or anti-mosquito infrastructural measures applied in houses (such as mosquito nets), as it is unpredictable which option people would choose, if any. We consider that emergency aerial spraying, even if proven to be effective in reducing mosquito populations and the number of human cases of WNV infection in the US [40], would not be the first option of vector control in Belgium, given the substantial environmental risks; furthermore, in our opinion, such a measure would not be easily accepted by the population. Also, the risk of mosquitoes developing resistance against insecticides should not be neglected. Furthermore, a recent study identified knowledge gaps concerning vector control in Europe and urged that the most appropriate and environmental friendly control strategies should be identified, given the reduced availability of products for mosquito control in recent years [54].

Neither outpatient costs (e.g. nursing home, transportation and child care) nor long-term costs (e.g. durable medical equipment, medication, medical appointments and institutional care) were taken into account, unlike previous estimations [35]. Actual costs might thus be higher. Inclusion of such costs would have been difficult, however, as it was a predictive study. Long-term costs of potential WNND after-effects, such as cost of treatment in rehabilitation facilities, were not evaluated, unlike a previous study in the US [53], as our scenario assumed the absence of after-effects in recovering patients (which is far from being the case when considering AFP).

The financial burden for the Belgian public health agency was assumed to be already included in its annual financing package. Communication about the epidemic by public agencies (e.g. production of brochures for the whole population or information aimed at horse owners) could be a cost to consider as well, but is difficult to estimate when taking a predictive approach, as it would probably be related to the importance of the epidemic.

The screening of blood and organ donors (most human infections being asymptomatic [4]) could be recommended, especially for those who return from an area with ongoing transmission of WNV in humans. Blood donations from WNV-positive donors should thus be deferred, as transmission of WNV through blood transfusion and organ donation has been well assessed [55]. The European Commission established a deferral period for prospective donors of 28 days after leaving an area with ongoing transmission of WNV in humans [56]. Blood and organ screening should be considered as an important contribution to epidemiosurveillance and epidemiovigilance systems [57] (useful to detect emergence of the disease). 

The US healthcare system is quite different from that in western Europe. In Belgium, *in the event of absence for* medical reasons, a worker (at least an employee) does not lose their salary: the loss is thus borne by the employer. Nevertheless, for long periods of illness, it is possible for an employer to hire interim staff, to replace the worker on sick leave. Thus productivity losses could be attenuated, even if a replacement contract generates additional costs for the employer (especially if they rely on employment agencies).

### Conclusion

The originality of our study is its prospective approach (preparedness) compared with previous works estimating economic impact of WNV in the US (retrospective studies). Our two scenarios relied on the variation of one parameter only, i.e. infection rate, while almost all parameters entered in the model are subject to uncertainty; a multifactor sensitivity analysis would have certainly widened the range of estimates. Better quality information is needed to predict the cost of a WNV outbreak in Belgium with more accuracy. In horses, if animal health authorities were to decide to recommend or to make vaccination compulsory, the choice of the strategy would have major consequences in terms of costs. Furthermore, animal health authorities would have to consider the delay involved in producing a high number of vaccine doses. It would thus not be possible to vaccinate the entire horse population during a first hypothetical epidemic. Targeted vaccination of horses at risk (living in habitat suitable for *Cx. pipiens*) could be a first-line preventive measure. A cost–benefit analysis of horse vaccination versus vector control is worth investigation. In humans, hospital charges would be the major financial consequences of an epidemic of WNV infection. It is thus essential to invest in research on preventive measures in the European context, e.g. through the development of a human vaccine (as none is commercialised to date) and integrated biological control of vectors, on a large scale. Integration of these impacts in healthcare plan/insurance schemes are of prime importance in terms of preparedness.
